# Clinical Utility of Serum Neurofilament Light Chain in Peripheral Neuropathy

**DOI:** 10.1002/mus.70073

**Published:** 2025-11-20

**Authors:** Chafic Karam

**Affiliations:** ^1^ Department of Neurology University of Pennsylvania Philadelphia Pennsylvania USA

**Keywords:** ATTRv, CIDP, CMT, hATTR, MAG, MMN, neurofilament light chain, peripheral neuropathy, vasculitic neuropathy

## Abstract

**Introductions/Aims:**

The clinical utility of serum neurofilament light chain (sNfL) in the evaluation and management of peripheral neuropathy (PN) remains poorly defined. This study aimed to evaluate the utility of sNfL for diagnosing PN, assessing disease activity, and monitoring treatment response using a commercially available assay.

**Methods:**

This was a retrospective cohort study at the University of Pennsylvania between June 2024 and March 2025. Patients with, or at risk for, PN who underwent sNfL testing were included. Demographics, PN etiology, clinical findings, and sNfL levels were analyzed.

**Results:**

One hundred and twenty‐eight patients were included: 41 with chronic inflammatory demyelinating polyneuropathy (CIDP), 36 with transthyretin amyloidosis (ATTR), 13 with vasculitic PN, 13 with Charcot–Marie–Tooth disease (CMT), 6 with multifocal motor neuropathy (MMN), 4 with anti‐MAG neuropathy, 4 with Guillain‐Barré syndrome (GBS), and 11 with other PN types. Of these, 113 had definite large fiber PN; 14 were asymptomatic TTRv carriers, and one had small fiber neuropathy. Elevated sNfL levels were observed in 31 patients (24%). The odds ratio of having elevated sNfL in treatable vs. non‐treatable PN was 28.33 (95% CI: 5.04–159.18). Among CIDP and ATTRv‐PN patients, sNfL was most often elevated in treatment‐naïve or refractory cases and decreased with treatment.

**Discussion:**

Routine sNfL testing is warranted in selected patients with PN, such as treatment‐naïve or refractory CIDP, active vasculitic PN, and ATTRv‐PN. Elevated sNfL in patients with PN should prompt evaluation for a potentially treatable cause and may offer useful adjunctive information to support clinical decision‐making.

## 1 Introduction

Serum neurofilament light chain (sNfL) has emerged as a promising biomarker for various neurological conditions, including peripheral neuropathy (PN) [1, 2, 3, 4, 5, 6, 7, 8, 9, 10, 11]. A recent systematic review and meta‐analysis suggested that blood neurofilament light chain may be a useful indicator of neuronal injury in PN [12]. However, the role of sNfL in the diagnosis and management of PN in clinical practice remains unclear.

This uncertainty exists despite a clear need: traditional tools for evaluating PN, such as clinical history, examination, and electrodiagnostic testing, can lack sensitivity and objectivity, particularly in early disease or when assessing ongoing activity. In theory, sNfL may offer a valuable adjunct, providing biologic insight into axonal damage. Importantly, sNfL testing has recently become commercially available, making its use in clinical settings increasingly feasible.

Yet the interpretation of existing studies remains challenging. Most prior work has involved comparisons of mean values of sNFL between patient groups and healthy controls. Many studies used blood bank samples without longitudinal follow‐up. Furthermore, there is often substantial overlap in sNfL levels between patients with neuropathy and controls, even with severe conditions such as Guillain‐Barré syndrome. Finally, nearly all published studies used the Quanterix Simoa assay, while commercially available testing now primarily relies on the Roche Diagnostics Electrochemiluminescence Immunoassay (ECLIA), which has distinct analytical characteristics.

As a result, clinicians are left with limited guidance on how to interpret sNfL values or integrate them into individual patient care. The aim of this study is to describe the experience using sNfL testing in routine clinical practice at a tertiary neuromuscular center in order to provide practical insight into how sNfL testing may (or may not) be helpful for diagnosing PN, gauging disease activity, and informing treatment decisions.

## 2 Methods

After obtaining approval from the University of Pennsylvania Institutional Review Board, a retrospective review of medical records of patients evaluated by the author between June 1, 2024, and June 30, 2025, was performed. Inclusion criteria were: (1) confirmed diagnosis of PN based on clinical evaluation and ancillary testing, OR carrier of a transthyretin pathogenic variant (ATTRv); (2) availability of serum neurofilament light chain (sNfL) measurement between June 2024 and March 2025; and (3) complete clinical documentation at the time of sNfL testing. Patients with isolated entrapment neuropathies were not considered to have PN. PN was defined as patients with symmetric or asymmetric length‐dependent PN, multifocal polyneuropathy or polyradiculoneuropathy, and those with non‐length‐dependent PN. The 2021 EAN/PNS Guideline on Management of Chronic Inflammatory Demyelinating Polyradiculoneuropathy was used for the diagnosis of patients with CIDP [13].

Demographic data, including age and sex, was collected. Neuropathy etiology was recorded when available. For hereditary neuropathies, genetic variant details were noted. Disease onset age was documented. Among transthyretin variant (TTRv) patients, clinical evidence of neuropathy or involvement of other organs, serum TTR levels, and biopsy results (when performed) were recorded. For vasculitic neuropathy patients, the vasculitic neuropathy subtype (systemic versus non‐systemic and primary versus secondary) [14, 15] and clinical assessment of neuropathy activity, based on worsening of the Neuropathy Impairment Score (NIS score) [16], was noted.

For patients with CIDP, multifocal motor neuropathy (MMN), and anti‐MAG neuropathy, disease onset, INCAT (Inflammatory Neuropathy Cause and Treatment) disability score [17], I‐RODS (Rasch‐built Overall Disability Scale) [18], grip strength, Timed Up and Go (TUG) [19], (NIS), as well as treatment regimen and dosages were documented. Electrodiagnostic findings were also collected. Patients with CIDP were considered treatment naïve if they had never been treated or had not received any treatment in the last 6 months. Patients were considered to be stable if there was no decline in their function, both subjectively and objectively. Objective assessments included INCAT, I‐RODS, grip strength, TUG, and neurological examination. They were considered refractory if they did not improve their function subjectively and objectively after adequate CIDP treatment (dose and duration).

Electrodiagnostic studies were performed by members of the Neuromuscular Division at the Hospital of the University of Pennsylvania Electrodiagnostic Laboratory. Skin temperature was maintained at least 33°C at the palm and 30°C at the external malleolus for NCV studies. Peroneal, tibial, ulnar, and median motor nerve responses were obtained when recording from the extensor digitorum brevis, adductor hallucis, abductor pollicis brevis, and abductor digiti minimi while stimulating at the distal leg, wrist, knee, and elbow. Sensory studies of the median, ulnar, radial, sural, and superficial peroneal nerves were performed antidromically using standard techniques [20]. Additional nerves were studied depending on the question to be answered. Patients were diagnosed with idiopathic polyneuropathy when they had a distal symmetric predominantly sensory polyneuropathy that was slowly progressive and had undergone screening for diabetes, paraproteinemia, vitamin B12 or B1 deficiency, vitamin B6 deficiency or toxicity, and a hereditary neuropathy panel. Social and medical histories were taken into account and additional testing was done accordingly.

Potential confounders affecting sNfL levels—such as age, diabetes status, kidney function, and body weight—were recorded. When available, treatment response and longitudinal sNfL changes were analyzed. Selected cases with notable clinical or biomarker changes were studied in detail.

Serum NfL measurements were performed by Labcorp (Burlington, North Carolina, USA) using the Elecsys Electrochemiluminescence Immunoassay (ECLIA) (Roche Diagnostics, Rotkreuz, Switzerland). Labcorp's normative data is based on over 120 samples for each age decade between 20 and 79 years and 40 samples for individuals aged 80 and above. According to Labcorp's internal validation, Roche Elecsys sNfL correlates strongly with the Quanterix Simoa (Billerica, Massachusetts, USA) assay (Deming Regression *R* ^2^ = 0.9924) [21]. Independent studies have also demonstrated a strong correlation between the Quanterix Simoa, the Roche Elecsys, the Siemens Healthineers AtellicaIM (Erlangen, Germany), and the Fujirebio Lumipulse (Tokyo, Japan) NfL assays, although with the Roche Elecsys assay, NfL concentrations are significantly lower (∼85%) when compared against the other three assays [22]. The test was performed only in patients who expressed interest in having their sNfL checked.

Log10 age‐adjusted sNfL levels were calculated using a linear regression model and compared using Mann–Whitney U test. sNfL levels before and after treatment in the ATTRv and CIDP patients were compared using a paired *t*‐test. Odds ratio of having elevated sNfL levels vs. normal sNfL levels was calculated comparing treatable causes of PN pre‐treatment (CIDP and ATTRv) vs. non‐treatable causes of PN (CMT, idiopathic, and remote CIPN patients). Excel (Microsoft, Redmond, WA) was used to collect the data, create the tables, and perform the statistical analyses.

## 3 Results

In the period studied, 432 patients underwent evaluation for PN. Of those,128 fulfilled the inclusion criteria. Among them, 41 had chronic inflammatory demyelinating polyneuropathy (CIDP), 4 GBS, 13 vasculitic PN, 6 multifocal motor neuropathy (MMN), 4 anti‐MAG neuropathy, 36 transthyretin‐related conditions (33 TTRv, 3 TTRwt, of whom 14 were asymptomatic carriers), 13 Charcot–Marie–Tooth disease (CMT), 5 idiopathic PN, 1 Fabry with SFN, 1 diabetic PN, 2 chemotherapy‐induced PN, 1 PN and MGUS, and 1 polyneuropathy, organomegaly, endocrinopathy, M‐protein and skin changes (POEMS). Of these, 113 had definite large fiber PN. Elevated sNfL levels were detected in 31 patients with PN (27% if not counting the TTRv carriers—none of the TTRv carriers had elevated sNFL). The odds ratio of having elevated sNfL in treatable vs. non‐treatable PN was 28.33 (95% CI: 5.04–159.18).

**FIGURE 1:**
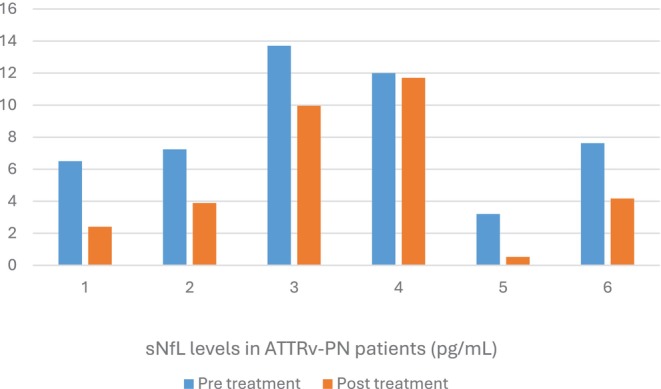
The figure shows sNfL levels in ATTRv‐PN patients who were treatment naïve and the change in levels after treatment. Note that only patients with elevated sNfL levels and follow‐up testing are shown. sNfL became normal in four out of the six patients treated.

Table 1 summarizes patients with TTR. Seven patients had elevated sNfL levels. Six patients who were treatment naïve had follow‐up levels after starting TTR silencer therapy. sNfL levels dropped in all but one of these patients (who was also the most advanced) (Figure 1).

**TABLE 1:** Summary of patients with TTR‐related disorder.

	TTRv no PN (14)	ATTRwt‐PN (3)	ATTRv‐PN treatment naïve (8)	ATTRv‐PN treated (11)
Sex (female/total)	11/14	0/3	2/8	4/11
Age (median, 25th–75th percentile)	58 (48.25–63.25)	73 (70.5–77.5)	69.5 (67.5–74)	67 (62–76)
Mutation	V122I (10) V30M (4)	N/A	V122I (2); V30M (4); T60A (1) Ala97Ser (1)	V122I (1); V30M (3); T60A (2); S77Y (1); 107 V (1); Phe4Leu (1); phe33ile (1)
CTS	5	3	5	8
Cardiac involvement	0	3	6	9
GI involvement	0	0	4	5
Ocular involvement	1	0	1	2
Autonomic involvement	0	0	4	4
Diabetes	3	0	0	1
Kidney failure	0	0	0	0
Obesity	0	0	0	0
sNfL (abnormal)	0	0	5	2
sNFL (median, 25th–75th percentile)	2.65 pg/mL (1.79–3.18)	2.47 pg/mL (2.45–3.1)	6.41 pg/mL (4.24–9.62)	4 pg/mL (2.77–4.66)
log10 sNfL (adjusted to age)	0.42 pg/mL (0.188–0.535)	0.393 pg/mL (0.389–0.483)	0.597 pg/mL (0.737–0.936)	0.602 pg/mL (0.435–0.663)
			p0.006 (sNfL levels comparison between treatment naïve and asymptomatic)	

**FIGURE 2:**
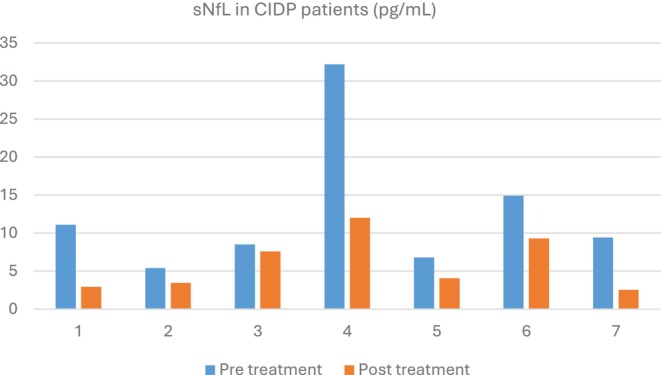
The figure shows sNfL levels in CIDP patients who were treatment naïve or refractory and the change in levels after treatment. Note that only patients with elevated sNfL levels and follow‐up testing are shown. sNfL levels became normal in four out of the six patients treated.

Table 2 summarizes patients with CIDP. Seven treatment‐naïve patients had follow‐up testing after treatment. Patients improved with IVIG, and sNfL improved in most (Figure 2). Three patients with stable disease had slightly elevated sNfL: one patient, age 49, had a level of 2.16 pg/mL (nL < 2.13), the other, age 29, had a level of 1.4 pg/mL (nL < 1.3), and the last, age 79, had a level of 7.74 pg/mL (nL < 7.64). The latter had diabetes. The ages of all these patients were within 1 year of transitioning to another decade that would have resulted in a different normative value, placing their results in the normal range. Alternatively, they could have mild, subclinial, active disease.

**TABLE 2:** Summary of CIDP patients.

	Treatment Naïve (pre‐treatment) (8)	Refractory (9)	Stable (24)	
Sex (female/total)	4/8	4/9	7/24	
Age (median, 25th–75th percentile)	53.5 (36.75–58.5)	59 (55–74)	65 (55–79)	
Typical CIDP	6	8	17	
Current treatment (IVIG vs. other)	0	2	21	
Off Treatment	8	2	1	
Diabetes	3	2	4	
Kidney failure	2	1	3	
Obesity	0	0	4	
INCAT	4 (2.25–4)	4 (2–5)	2 (1–4)	
sNfL (abnormal)	7	3	3	
sNFL (median, 25th–75th percentile)	9.63 pg/mL (6.95–15.2)	5.39 pg/mL (1.99–6.79)	1.87 pg/mL (1.4–3)	
log10 sNfL (adjusted to age)	0.957 pg/mL (0.688–1.105)	0.284 pg/mL (0.641–0.917)	0.272 pg/mL (0.174–0.479)	p0.0007 treatment naïve vs. stable

Table 3 summarizes the rest of the patients studied. In one patient with active vasculitic PN, the sNfL dropped from 7.56 to 2.03 pg/mL (normal < 2.99) after treatment.

**TABLE 3:** Summary of the patients with other forms of PN.

	Vasculitis	CMT	MMN	GBS	MAG	Other
Sex (female/total)	5/13	9/13	2/6	2/4	2/4	5/11
Age (median, 25th–75th percentile)	67 (53–69.5)	63 (46.5–70.5)	60 (45.5–73.5)	52.5 (39.7–63.7)	75.5 (67.5–77.5)	63 (53–68)
Diabetes	1	1	0	0	0	1
Kidney failure	2	0	0	0	0	1
Obesity	2	0	0	0	0	6
sNfL (abnormal)	3	2	0	3	0	0
sNFL (median, 25th–75th percentile)	2.8 pg/mL (1.37–4.57)	2.26 pg/mL (1.97–2.97)	2.12 pg/mL (1.4–3.3)	3.58 pg/mL (2.2–120.6)	3.05 pg/mL (2.09–3.83)	2.67 pg/mL (1.82–4.18)
log10 sNfL (adjusted to age)	0.441 pg/mL (0.091–0.652)	0.354 pg/mL (0.292–0.482)	0.315 pg/mL (0.132–0.475)	1.401 pg/mL (0.389–0.55)	0.390 pg/mL (0.239–0.541)	0.396 pg/mL (0.183–0.620)

More details on the patients studied can be found in the Supporting Information.

## 4 Discussion

This study indicates that serum neurofilament light chain (sNfL) may provide value in select patients, particularly when levels are elevated. Along those lines, elevated sNfL in patients with PN should prompt evaluation for a potentially treatable cause. On the other hand, a normal sNfL should not give a false sense of reassurance or imply that the neuropathy is inactive. Based on the findings of this study and review of the literature, measuring sNfL in patients with PN may be most useful in the following situations:

In patients with GBS, new onset CIDP, and those with refractory CIDP or concern about optimal treatment, or when a treatment change is being considered. GBS is a monophasic illness. The sNfL is elevated in many patients with GBS at the time of diagnosis, peaks at 14 days, and then starts decreasing [2]. While sNfL levels in GBS can be very elevated in comparison to CIDP and other neuropathies studied here, [3, 4, 5, 8] some patients with GBS may have normal sNfL levels [1]. This could be explained by the fact that sNfL levels peak at about 2 weeks in GBS, and that in some milder cases, and possibly mostly in demyelinating disease, the sNfL may be normal early in the course [2]. Interpreting serial measurements can be challenging given the unclear half‐life of sNfL (some have estimated it at around 500 h) [23]. In CIDP, sNfL levels were particularly useful for identifying active disease and monitoring treatment responses. For instance, sNfL levels were elevated in untreated patients and those with inadequate treatment, reflecting ongoing disease activity, and they generally improved with successful treatments [3, 4, 8]. No major conclusions can be made regarding MMN, MAG, and POEMS based on this study. None of them had elevated sNfL, but most were treated. Only one MAG patient was treatment naïve. Studies in MMN have mainly focused on differentiating MMN from motor neuron disease, with levels typically much higher in the latter [7, 8]. In MAG, a large study showed that sNfL was overall normal, but another smaller study suggested that the levels were elevated [9, 24]. There was only one patient with POEMS who was treated. Untreated patients could have elevated sNfL levels similar to CIDP. Other biomarkers, such as plasma periaxin, may be important biomarkers in peripheral nerve demyelination [25].

In patients with ATTRv‐PN. many of the treatment‐naïve patients with ATTRv‐PN had elevated sNfL levels. sNfL tended to drop with treatment and sometimes normalize [26]. Although some studies have suggested that sNfL can be an objective measure to detect the transition from the presymptomatic stage to the onset of symptomatic disease, in presymptomatic carriers, some of our patients with definite ATTRv‐PN had normal sNfL. In the absence of symptoms and signs of neuropathy, it is unlikely that the sNfL would be elevated [26, 27].

In vasculitic neuropathy, the role of sNfL may have value in treatment‐naïve patients or in those who are not sufficiently treated [6]. Only one patient in the latter group and none in the former were included in this study. Only one study has looked at a series of patients with untreated vasculitic neuropathy, and most had elevated sNfL levels. Levels decreased with treatment, and in two patients who had relapse, the levels increased again [6].

In CMT and other neuropathies (idiopathic, MGUS, diabetic, Fabry, and remote history of chemotherapy‐induced PN), the role of sNfL in clinical care is less clear. Only 2 patients with CMTx had moderately elevated sNfL, and the rest of this group of patients had normal sNfL. Some studies have shown that, as a group, patients with CMT1a, CMTx, and CMT2A had elevated sNfL compared to controls, but other subtypes of CMTs had normal sNfL [28]. One could suspect that some CMT patients who develop superimposed CIDP may have elevated sNfL. In those, there may be utility in checking sNfL, but this has not been studied. In chemotherapy‐induced PN (CIPN), it has been shown that sNfL increases in the acute setting, as was shown in this study [29]. None of the idiopathic PN patients had elevated sNfL.

It is important to stress a few important observations: (1) sNfL levels should always be interpreted along with the history, the patient's reported symptoms, clinical examination, and, if indicated, electrodiagnostic testing and other laboratory testing. (2) sNfL is not specific to PN, and other neurological disorders or factors (such as kidney failure) can affect the results. (3) sNfL may not be very sensitive in slowly progressive polyneuropathies, even if autoimmune or affecting mainly the myelin or node of Ranvier‐ hence normal levels should not dissuade the clinician from prescribing disease‐modifying therapy if they believe it is clinically indicated. (4) It is unclear how one should change therapy if sNfL remains elevated, especially in advanced ATTRv‐PN patients. Finally, one needs to keep in mind the cost of these tests.

Limitations of this study include the small number of patients with sNFL tested before initiation of treatment when treatment was available, the lack of prospective data in some of the patients of interest, and the small number of patients with some disorders, such as POEMS, MAG, MMN, and wtTTR.

In conclusion, sNfL testing may be helpful in selected clinical scenarios—particularly when clinicians are faced with diagnostic uncertainty, are considering escalation or de‐escalation of treatment, or need additional data to evaluate disease activity in conditions such as CIDP, GBS, vasculitic neuropathy, and ATTRv‐PN. In addition, elevated sNfL levels may warrant further investigation for an underlying treatable etiology. Further prospective and longitudinal studies are needed to clarify the utility of sNfL in various neuropathies, in particular POEMS, MAG, and MMN. Ultimately, sNfL is probably best used as a complementary tool alongside a comprehensive clinical evaluation.

## Author Contributions

**Chafic Karam:** conceptualization, investigation, writing – original draft, data curation, formal analysis.

## Funding

The author has nothing to report.

## Ethics Statement

I confirm that I have read the Journal's position on issues involved in ethical publication and affirm that this report is consistent with those guidelines.

## Conflicts of Interest

I have served as a consultant and/or advisory board: Acceleron, Alpine, Alexion, Alnylam, Amgen, Amicus, Annexon, Argenx, Astra Zeneca, Corino, Biogen, CSL Behring, Genentech, Ionis, J&J, Neuroderm, Novo Nordisk, Octapharma, Pfizer, Sanofi, UCB, Takeda, Vertex, and Zai lab. I have also obtained research funding from Argenx, Ionis and Genzyme.

## Supporting information


**Data S1:** mus70073‐sup‐0001‐Supinfo.docx.

## Data Availability

The data that support the findings of this study are available from the corresponding author upon reasonable request.
