# Supplementary material for: Clinical Utility of Serum Neurofilament Light Chain in Peripheral Neuropathy
Source: Muscle Nerve. 2025 Nov 20;73(1):86–92. doi: 10.1002/mus.70073 (PMC12690012; doi:10.1002/mus.70073)
Supplement: Supplementary file 1 — Data S1: mus70073‐sup‐0001‐Supinfo.docx. [file MUS-73-86-s001.docx]

Supplemental material

**Description of work-up and findings in ATTRv no PN (table 1).**

All TTRv no PN had some vague neurological symptoms prompting evaluation. They all underwent extensive neurological assessment, including nerve conduction studies, autonomic testing, and skin biopsies. None had evidence of PN on nerve conduction studies or skin biopsy. Autonomic reflex screens were normal in all but one patient. That patient had reduced quantitative sweat response at the foot, which, in isolation, was felt to be of unclear clinical significance. There was no amyloid deposition in the skin. Additional gastrointestinal biopsies and fat aspirate were also conducted in 3, and were negative for amyloid deposition. All patients underwent cardiac work-up, including Pyrophosphate (PYP), and did not have cardiac amyloidosis. One patient had evidence of ocular amyloid deposition based on Optical Coherence Tomography (OCT).

**Details of refractory CIDP patients (table 2)**

Patients were considered refractory if they did not improve with first-line therapy (steroids, IVIG, and plasma exchange (PLEX)). Six responded to additional therapy (usually rituximab or cyclophosphamide, and one underwent a stem cell transplant. The other 3: 1 patient with motor CIDP initially responded to steroids and IVIG, but relapsed. An increase in IVIG and the addition of rituximab resulted in improvement. A year later, the patient decided to stop treatment with rituximab; at that time, sNfL was normal, but upon follow-up, the patient's symptoms worsened and sNfL increased (9.43 (nl<7.64 pg/mL). After treatment, he improved, and SNfL decreased (2.54pg/mL). The second patient was off any therapy for a year. Disease activity was uncertain. Baseline sNFL was elevated. Rituximab was started, and the patient improved. sNfL level normalised supporting clinical impression. However, additional rituximab was denied by insurance, and 3 months later, the patient worsened again, and sNfl went back up. The last patient had an incomplete response to IVIG (INCAT 5 to 3), and sNfl remained elevated. An increase in IVIG dose did not help. sNFL remained elevated. Adding IV solumedrol weekly for 3 months resulted in improvement in strength on examination and reduction in sNfL, but not normalisation. The patient did not subjectively feel better.

**Details of patients with other forms of polyneuropathy (table 3)**

1. Five isolated PNS vasculitis, 1 cryoglobulinemia, 3 ANCA-associated vasculitis, 1 RA, and 1 SS all on treatment. One Eosinophilic Granulomatosis with Polyangiitis (EGPA), partially treated. With rituximab, sNfL normalized. The other had multiple myeloma worsening neuropathy and sNfL after daratumumab, cyclophosphamide, bortezomib, and dexamethasone (Dara-CyBorD). Neuropathy and sNfL improved after stopping bortezomib. The third had isolated PNS vasculitis in remission, elevated sNfL related to acute stroke.
2. 3 CMT1a, 2 CMT1b, 3CMTx, 1CMT1c, 1 CMT2c, 1CMT2, 1 CMT2U, 1 CMT4c. sNfL levels elevated in 2 CMTx female, normal in male patient.
3. All stable on IVIG
4. One GBS patient had the highest sNfL of the group studied, by far (120.6pg/mL). He was tested 1 week after symptoms onset. 3 months later levels were 4.91pg/mL (nl < 3.78). The other 2 were tested 2 months after the GBS and had slightly elevated levels. The last one had normal level 6 months after GBS.
5. One not treated, with slowly progressive disease; the other 3 were treated (2 Rituximab and 1 Zanutrinib and Rituximab).
6. Six with idiopathic PN (one had MGUS and CKD), 1 treated Fabry female patient, 1 treated POEMS, 2 remote CIPN, and 1 diabetic PN.

**Description of the ATTRv-PN patients who had available sNfL levels before and after treatment (Figure 1)**

Patient 1 is a 67-year-old man with V30M TTRv with PN (NIS 22), orthostatic hypotension, and bilateral carpal tunnel syndrome. PYP was positive, but he had no heart failure.

Patient 2 is a 69-year-old man with T60A TTRv with PN (NIS 4) and heart failure.

Patient 3 is a 68-year-old man with Ala97Ser TTRv with PN (NIS 78), dysautonomia, and bilateral carpal tunnel syndrome. No cardiac involvement.

Patient 4 is a 75-year-old man with V30M TTRv with PN (NIS 86), bilateral carpal tunnel syndrome, and heart failure.

Patient 5 is a 46-year-old man with Phe33ile TTRv with PN (NIS 8), and ocular amyloid. No cardiac involvement.

Patient 6 is a 62-year-old man with phe33ile TTRv with PN (NIS 28), and bilateral CTS. PYP was positive, but he had no heart failure.

**Description of the CIDP patients who had available sNfL levels before and after treatment (Figure 2)**

Patient 1 is a 54-year-old man with typical CIDP, not responsive to IVIG but responsive to steroids. INCAT before treatment was 4 and decreased to 2 after treatment.

Patient 2 is a 53-year-old woman with typical CIDP responsive to IVIG. INCAT before treatment was 5 and decreased to 2 after treatment.

Patient 3 is a 33-year-old woman with distal CIDP responsive to IVIG. INCAT before treatment was 4 and decreased to 2 after treatment.

Patient 4 is a 63-year-old man with typical CIDP responsive to IVIG. INCAT before treatment was 3 and decreased to 2 after treatment.

Patient 5 is a 54-year-old man with an acute onset of CIDP responsive to IVIG. INCAT before treatment was 4 and decreased to 2 after treatment.

Patient is a 60-year-old woman with focal onset CIDP. INCAT before treatment was 1 and decreased to 0 after treatment.
